# Effects of tai chi on cognition and instrumental activities of daily living in community dwelling older people with mild cognitive impairment

**DOI:** 10.1186/s12877-018-0720-8

**Published:** 2018-02-02

**Authors:** Mei-yi Siu, Diana T. F. Lee

**Affiliations:** 1 The School of Nursing, Tung Wah College, 31 Wylie Road, Homantin, Kowloon, Hong Kong, SAR People’s Republic of China; 2 The Nethersole School of Nursing, Chinese University of Hong Kong, Hong Kong, SAR People’s Republic of China

**Keywords:** Tai chi, Cognition, Instrumental activities of daily living, Mild cognitive impairment

## Abstract

**Background:**

Cognitive impairment places older adults at high risk of functional disability in their daily-life activities, and thus affecting their quality of life. This study aimed to examine the effects of Tai Chi on general cognitive functions and instrumental activities of daily living (IADL) in community-dwelling older people with mild cognitive impairment (MCI) in Hong Kong.

**Methods:**

The study adopted a multi-site nonequivalent control-group pretest-posttest design. 160 community-dwelling older people, aged ≥60, with MCI, from four community elderly centers participated in the study. The intervention group (IG, *n* = 80) received training in the Yang-style simple form of Tai Chi, at a frequency of two lessons per week for 16 weeks. Each lesson lasted for one hour. The control group (CG, *n* = 80) had no treatment regime and joined different recreational activity groups in community centers as usual within the study period. Outcome measures included measures of global cognitive status and IADL. The Chinese version of the Mini-Mental State Examination (CMMSE) was used for global cognitive assessment. The Hong Kong Chinese version of Lawton’s Instrumental Activities of Daily Living (IADL-CV) was used to assess the participants’ IADL levels. General Estimating Equations (GEE) was used to examine each of the outcome variables for the two groups at the two study time points (the baseline and at the end of the study). Meanwhile, minimum detectable change (MDC) was calculated to estimate the magnitude of changes required to eradicate the possibility of measurement error of outcome measures.

**Results:**

Seventy four participants in the IG and 71 participants in the CG completed the study. With adjustments for differences in age, education, marital status and living conditions, the findings revealed that the participants in the IG scored significantly better on the CMMSE test (*P* = 0.001), and the instrumental ADL questionnaire (*P* = 0.004). However, those scores changes did not exceed the limits of the respective MDCs in the study, the possibility of measurement variation due to error could not be excluded.

**Conclusion:**

Tai Chi may be an effective strategy to enhance cognitive health and maintain functional abilities in instrumental ADL in older people with MCI.

**Trial registration:**

NCT03404765 (Retrospectively registered January 19, 2018)

**Electronic supplementary material:**

The online version of this article (10.1186/s12877-018-0720-8) contains supplementary material, which is available to authorized users.

## Background

As ageing population is rising all over the world, age-related cognitive decline has been highlighted as a public health concern worldwide. Nowadays, 47.5 million people worldwide live with dementia, and there are 7.7 million new cases every year. These figures are projected double every twenty years, reaching 75.6 million in 2030 and 135.5 million in 2050 [1]. In the Asia Pacific region, people diagnosed with dementia are expected to increase from 27 million in 2015 to 70 million by 2050 [2].

The aging population in Hong Kong is largely a consequence of a longer life expectancy and lowering fertility rate. According to the Census and Statistic Department (2015), the proportion of elderly citizens aged 65 or above reached 15% in 2014 and is projected to rise markedly to 33% in 2064 [3]. Thus, dementia occurred in 9.3% of people aged 70 and above in 2008 and is expected double for every 5 years of increase in age [4]. The prevalence rate of dementia is projected to increase by 222% from 2009 to 2039 [5].

With increasing awareness about the importance of promoting cognitive health in the aging population, mild cognitive impairment (MCI) has become a major research topic in the field of dementia and related cognitive disorders. MCI has been described as an intermediate stage of cognitive deterioration, from normal cognition to fully developed symptoms of dementia [6]. Persons with MCI pose with greater risk of developing dementia [7].

### Cognitive and functional deficits in mild cognitive impairment

Cognitive and functional impairments are hallmark features to distinguish the difference between MCI and dementia. Although the general cognitive functions and basic activities of daily living (BADL) such as bathing, continence, dressing, feeding, toileting and transferring, are largely preserved, numerous researchers have suggested that people with MCI may experience some deficits in performing instrumental activities of daily living (IADL), including the ability on using telephone, shopping, preparing food, doing house chores and laundry, using transportation, self-medication, and managing financial affairs etc., comparing to age-matched cognitively healthy controls [8, 9]. IADL enactment must draw multiple cognitive domains for competent performance. Subtle difficulties illustrated by slowness and hesitation in carrying out complex ADL tasks may indicate some degree of dysfunction [10–14]. Highly cognitively demanding IADL was found to be positively correlated with performance in six cognitive domains, namely memory, attention, processing speed, executive function, language, and visuospatial ability [13]. Thus, MCI plus IADL limitations are associated with higher conversion and shorter duration to clinical manifestation of dementia [15–17].

While people with MCI have high chances of becoming demented, the heterogeneous nature of MCI may carry an unstable prognosis. MCI is not necessarily a prodromal syndrome of dementia, as many people not showing deterioration but become static in the status of their cognitive deficits, and in some cases, may revert to normal cognition [18, 19]. Persons with MCI may benefit from early interventions by slowing down the rate of progression to dementia.

### Tai chi as a strategy against cognitive and functional declines

At present, there is no consensus among medical scientists on any kind of drug treatment for people with MCI. Clinical trials of drugs used for the treatment of Alzheimer’s disease have shown no beneficial effects on delaying progression from MCI to dementia [20–22]. Furthermore, these cognitive-enhancing drugs can cause gastro-intestinal disturbances [22].

Research on non-pharmacological intervention, however, has suggested that lifestyle factors can help prevent dementia. Fratiglioni, Paillard-Borg, and Winblad (2004) identified three lifestyle factors that may contribute to slowing down cognitive decline, which included integrated social networking, cognitively stimulating activity, and regular physical activity [23]. An epidemiological study of elderly persons in Britain, with 21 years’ follow up, observed that both cognitive and physical activity had protective effects against cognitive decline [24]. Another retrospective cohort study in Dijon and Montpellier (France), analyzed lifestyle data from 5698 non-demented older citizens from 1999 to 2001. The result also suggested that cognitively stimulating leisure activities might delay the onset of dementia in community-dwelling older people [25].

Mind-body exercise, such as Tai Chi, is a type of physical exercise that combines physical and cognitive-stimulating activity. Tai Chi focuses on tranquility of mind to achieve longevity through meditation and life style modification [26]. It is an alternate form of aerobic exercise with moderate intensity [27]. Aerobic exercise can delay age-related brain atrophy, increase cerebral blood circulation, and stimulate neural cell regeneration [28, 29]. As an activity to promote cognitive stimulation, the meditation component of Tai Chi can improve attentional focus and executive function through learning a series of choreographed movements in continual sequence [30, 31]. As well, engaging in Tai Chi exercise in group settings allows elderly persons to maintain social contact with others, which can further benefit cognition through stress reduction and peer group support [32, 33].

Systematic reviews and meta-analysis studies have reported Tai Chi training can improve general cognitive performance, memory, attention, language, and executive functions in older adults, including people with MCI [34–37]. Effective management of IADL is dependent on memory, attention, and executive functions. [12, 13, 38]. Tai Chi can be conceived as an appropriate exercise for elderly persons to preserve cognitive health and functional abilities.

As aging populations increase rapidly all over the world, interventions to maintain cognitive functioning in elderly people will be of paramount important to preserve functional ability, independence, and HRQOL [34]. Cognitive impairment is one of the strong determinants of functional disability [39, 40]. Currently, there is limited research evidence to support the effects of Tai Chi training on IADL performance. Therefore, the aim of this study is to examine the effects of Tai Chi on general cognitive functions and instrumental activities of daily living (IADL) in community-dwelling older people with mild cognitive impairment (MCI) in Hong Kong.

## Methods

### Study design

This was a quasi-experimental study which adopted a multisite nonequivalent control group pre- and post-test design (Clinical trial registry: CREC Ref. No.:2015.348).

### Participants and settings

Convenience sampling was used to recruit participants from four community elderly centers. These elderly centers are run by non-government organizations which are registered under and monitored by the Social Welfare Department of Hong Kong. There are around 50 community elderly centers in Hong Kong. To gain access to the target population for this study, various elderly centers in different regions in Hong Kong were contacted. Each elderly center in the study has an enrollment of 200 to 300 members. They all operate with similar settings. Two of these centers were randomized as intervention groups, and the other two were treated as control groups. Recruitment process started after center assignment, and the participants were allocated to the experimental or control groups according to the centers to which they belonged. This arrangement avoided possible contamination of the experiment by diffusing the Tai Chi learning.

The domains of cognitive defect experienced by individuals with MCI vary. As the sample population in the study might comprise of different subtypes of MCI persons, the general cognitive function, as measured by the Chinese-Mini Mental State Examination (CMMSE) [41], was chosen as a screening criterion and an outcome variable for this study.

The inclusion criteria for the study were: (1) Chinese people aged 60 years or above; (2) the CMMSE screening score ranging from 19 to 28, which was corrected based on educational level (≥ 18 for illiterate respondents and ≥22 for those having received more than two years of schooling) [42]. The CMMSE score of 19 was set as the bottom limit because previous studies have found that older adults with MMSE scores of 18 can provide valid answers to inquiries and are able to follow instructions [43, 44]. At the upper limit, some elderly persons with MMSE scores of 28 may exhibit symptoms of memory deficit [45]; (3) ability to perform self-care functions on their own; (4) no confirmed diagnosis of dementia, depression or other psychiatric illnesses which would interfere with cognitive performance; and (5) no engagement in any structured physical exercise program or Tai Chi practice in the preceding year.

Elderly persons were excluded from the study if they: (1) had a medical history of chronic alcoholism or brain trauma occurred in previous years that could interfere with cognition; (2) had been regular users of medications that could affect cognition; or (3) had clinical conditions that contraindicated light to moderate physical exercise.

The participants were requested not to join any structured exercise training other than the Tai Chi interventions during the study period.

### Sample size estimation

A medium effect size of 0.5 was set for the study, based on the previous research on the effect of Tai Chi on cognitive function [46]. With the use of a sample size calculator (https://www2.ccrb.cuhk.edu.hk/stat/Other.htm), a sample of 64 per group was expected to provide 80% power to detect a medium effect size of 0.5, at a significance level of 0.05 [47]. Allowing for an attrition rate of 20% in the Tai Chi groups [46, 48, 49], 77 participants of each group would be needed to maintain sufficient power in the current study. To round up, 80 participants were recruited for each group.

### Intervention protocol

#### Intervention group

The intervention group (IG) received a 16-week Tai Chi program, of 32 sessions (2 sessions per week), each being one-hour long. The capacity was around 20 people per class. A certified Tai Chi master taught the Yang-style simple form of Tai Chi to the participants allocated to the IG. This Yang-style form was designed by a group of Tai Chi experts from the Chinese Sports Commission in 1956, and based on the Yang Tai Chi long form. It is sometimes called the Beijing or Peking form of Tai Chi, or the Tai Chi 24 form, because it consists of 24 unique movements [50]. There were four sets of movements included in the simplified form, that lesser time was required for the elderly practitioners, who usually had less exercise endurance, to complete the whole set of movements. Moreover, it was an appropriate form of Tai Chi for elderly persons with MCI because it involved only a few postures, and hence it was easier for them to learn and remember (Additional file 1: Table S1). The Tai Chi classes were conducted in the open hall of the community center or a public playground with sufficient space, a flat floor and good lighting.

Each Tai Chi session had three parts. The first part was the “warm up”, which was comprised of simple motions to help the elderly learners to relax their muscles and joints. The second part was “Tai Chi instruction”. The Tai Chi instructor taught the whole set of unique movements of the Yang-style simple form of Tai Chi to the groups, instructed them to move in circular motions with low speed and explained how to focus on breathing and muscle coordination. The final part was the “cool down”, which involved activities to cease the Tai Chi and rest the body.

To ensure adherence, the participants were required to sign attendance records. Telephone follow-up was arranged to provide emotional support, reinforcement and compliance if they were absent from two consecutive lessons. As well, the participants were advised to do daily practice at home to foster better mastering of the Tai Chi movements and build up the regular exercise habit. A guide book was issued to each participant, providing guidance to reinforce correct postures. As it was difficult to ensure the accuracy of participants’ self-reporting of their home practices, the out-of-class practice records served only as a source of communication to reinforce compliance with the Tai Chi practice.

#### Control group

The control group (CG) was treated as the usual care group and no exercise training was arranged. They were advised to attend different kinds of recreational activities provided by their elderly centers and to continue their daily activities, including their usual general physical mobility and social activities during the study period.

### Outcome measures

#### The Chinese version of mini-mental state examination (CMMSE)

The CMMSE is a global cognitive assessment tool which has been translated from the original MMSE version developed by Folstein, Folstein, and McHugh, in 1975 [51]. The CMMSE is divided into two parts. The first part of the instrument assesses respondent’s abilities in orientation, memory, and attention. The second part is to test respondent’s abilities in naming objects, understanding verbal and written commands, repeating short sentences, and drawing overlapping polygons [41].

The sensitivity and the specificity of cut-off score of the CMMSE at 19/20 were 97.5% and 97.3% respectively. The test-retest reliability of the instrument was 0.78 and the inter-rater reliability was 0.99 [41]. The CMMSE score ranges from 0 to 30; the higher the scores, the better cognitive performance implies.

#### The Hong Kong Chinese version of Lawton’s instrumental activities of daily living (IADL-CV)

The Hong Kong Chinese version of Lawton’s Instrumental Activities of Daily Living (IADL-CV) [52] is translated from the original English version of Lawton’s IADL scale [53]. Nine domains of IADL are assessed, including ability of using telephone, shopping, preparing food, doing house-keeping and laundry tasks, using transportation, managing finances, handling medication, and doing handyman work. The inter-rater and the test-retest reliabilities of the scale were 0.99 and 0.90 respectively. The Cronbach alpha assessing internal consistency was 0.86. The higher the scores of each domain, the better the functional abilities of the elderly persons to live independently in the community represent [52].

### Data collection process

All participants, both in the CG and the IG, received pre-study and post-study assessments based on identical sets of questionnaires. Demographic data and the baseline measurements for both groups were collected at the beginning of the study (T0). Post-study assessment (T1) was done 1–2 weeks after completion of the Tai Chi intervention.

### Data analysis

The IBM SPSS Version 24.0 (IBM Crop. Armonk, N.Y.) was used for data entry and analysis. All statistical tests involved were 2-tailed and the level of significance was set at 5%. Descriptive statistics, including mean, standard deviation, frequency, and percentage, were used to summarize and present the sample characteristics and outcome measures.

Chi-square, Fisher’s exact, and independent t- tests were used, as appropriate, to compare the characteristics of IG and CG at the baseline. If there were significant differences between two groups of these characteristics, they were adjusted in the subsequent outcome analysis. Standard error of measurement (SEM) and minimum detectable change (MDC) were calculated to estimate the magnitude of changes needed to confidently rule out the possibility of change in outcome measures owing to measurement error.

Generalized Estimating Equations (GEE) was applied for comparing each of the repeated measures study outcomes (i.e. CMMSE score, IADL-CV score) between the two groups with adjustment for those demographic characteristics showing statistical incomparability between groups. Two dummy variables, Group and Time, were included in each GEE model to represent the baseline group difference (Intervention – Control) and the time effect on the control group (Post 16 weeks – Baseline), respectively. Furthermore, the interaction term, Group by Time (Group*Time) was also included to assess the differential change in each outcome between the two groups. GEE model could account for intra-correlated repeated measures outcome and produce unbiased effect estimated under the data missingness mechanism of missing completely at random (MCAR).

### Ethical considerations

Approval for conducting the study was obtained from the Joint CUHK-NTEC Clinical Research Ethics Committee of The Chinese University of Hong Kong and the selected community elderly centers.

The participants were required to sign a consent form. Information and the purpose of the study were explained. They could withdraw from the study at any time of their own will.

## Results

The study was conducted from December 2015 to September 2016. A total of 178 elderly persons was screened for this study. Out of the 87 elderly persons (48.9%) screened for the IG, seven (8.0%) were rejected after the initial screening process. Of the 91 elderly persons (51.1%) screened for the CG, 11 (21.1%) were rejected. For the 18 rejected, 11 had scored very low marks in the CMMSE screening and the other seven were doing regular exercise. Eventually, there were 80 participants eligible for the IG and another 80 were admitted to the CG.

**Fig. 1:**
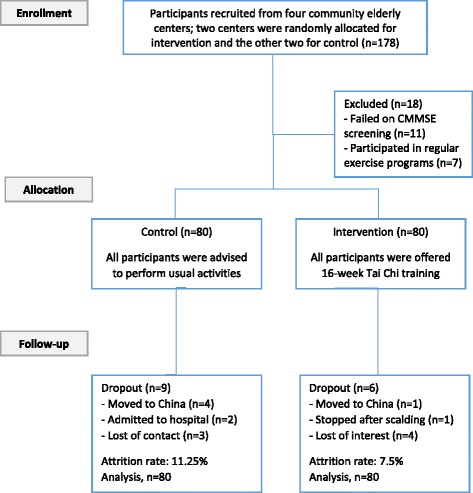
Flowchart to Show the Recruitment and Dropout of Participants in the Intervention and Control Groups

Throughout the study, there were six and nine participants dropped out for various reasons in the IG and CG respectively. The dropout rates were 7.5% for the IG and 11.25% for the CG (Fig. 1). The demographic characteristics of the completers and non-completers were basically comparable, and no significant difference was found.

The average attendance rate of all participants in the IG was 81.4%. Reasons for not attending the Tai Chi classes were mainly due to attending medical follow-up, not feeling well or not being suitable to carry out outdoor exercise in poor weather conditions. There was no reason for missing classes due to any discomfort after practicing Tai Chi. No injury report was obtained during the Tai Chi intervention.

73.8% female (*n* = 118) to 26.3% male (*n* = 42). A baseline demographic assessment showed that there was no significant difference between the two groups for gender (*P* = 0.72) or comorbid conditions (*P* = 0.278). However, significant differences between groups were observed in some of the demographic variables, including age, education level, marital status and living conditions. The participants in the IG were younger (*P* = 0.001), with higher academic attainment (*P* = 0.004) than those in the CG. However, there were more participants in the CG than in the IG who lived alone (*P* = 0.013) and reported their status as widowed/single/divorced (*P* = 0.05) (Table 1).

**Table 1:** Baseline demographic characteristics of the participants

Demographic characteristics	Overall (*n* = 160) Frequency (%)	Control (*n* = 80) Frequency (%)	Intervention (*n* = 80) Frequency (%)	Chi-Square (*X*^2^)	*P*-value
Sex				0.13	0.719
- Male	42 (26.3)	20 (25)	22 (27.5)		
- Female	118 (73.8)	60 (75)	58 (72.5)		
Age				10.19	0.001*
- 60–74	107 (66.9)	44 (55.0)	63 (78.8)		
- 75 or above	53 (33.1)	36 (45.0)	17 (21.3)		
Education Level				8.23	0.004*
- Illiterate or below primary school	70 (43.8)	44 (55.0)	26 (32.5)		
- Completed primary school or above	90 (56.3)	36 (45.0)	54 (67.5)		
Marital Status				7.76	0.005*
- Married	101 (63.1)	42 (57.5)	59 (73.8)		
- Single/ Widowed/ Divorced	59 (36.9)	38 (47.5)	21 (26.3)		
Living Condition				6.18	0.013*
- Living alone	35 (21.9)	24 (30.0)	11 (13.8)		
- With family members	125 (78.1)	56 (70.0)	69 (86.3)		
Comorbidity					0.278
- 0	33 (20.6)	14 (17.5)	19 (23.8)		
- 1–2	100 (62.5)	49 (61.3)	51 (63.8)		
- 3 or more	27 (16.9)	17 (21.3)	10 (12.5)		

*p≤0.05

The mean scores of CMMSE and IADL-CV for both CG and IG at the baseline and after 16 weeks of intervention, as well as their mean change scores are shown in Table 2. The IG showed greater improvement in both outcomes than the CG.

**Table 2:** Outcome variables across time between the study groups

Outcome variables	Control (n = 80)			Intervention (n = 80)		
	Mean (SD)	SEM	MDC (95%CI)	Mean (SD)	SEM	MDC (95%CI)
CMMSE						
- Baseline	24.61 ± 2.75	1.29	3.58	25.46 ± 1.89	0.89	2.46
- Post 16 weeks	24.70 ± 2.90			26.74 ± 2.42		
- Change of score	0.11 ± 2.78			1.38 ± 2.22		
IADL-CV						
- Baseline	24.20 ± 2.55	0.81	2.25	25.03 ± 1.76	0.56	1.56
- Post 16 weeks	23.96 ± 3.01			25.88 ± 1.65		
- Change of score	−0.21 ± 2.69			0.84 ± 1.82		

*Abbreviations*: *CMMSE* Chinese version of Mini-Mental State Examination, *IADL-CV* Hong Kong Chinese version of Lawton Instrumental Activities of Daily Living scale

General Estimating Equations (GEE) were used to compare the differential changes between groups in the outcome variables after 16 weeks of intervention, with adjustments for the demographic variables which were shown to be statistically incomparable, including age, education level, living conditions and marital status. After adjusting the potential confounding factors, the IG showed significantly greater improvement in CMMSE score than the CG, over the 16-week study period (group by time interaction, *B* = 1.33, 95% CI 0.53–2.13, *P* = 0.001; Cohen’s *d* effect size 0.50). Moreover, the IG revealed a significantly better improvement in IADL scores. The group by time interaction term was statistically significant (*B* = 1.07, 95% CI 0.34–1.81, *P* = 0.004; Cohen’s d effect size 0.48) (Table 3). Although participants of the IG demonstrated significantly greater improvements in both CMMSE and IADL-CV scores than the CG throughout the post-pre-study period, such changes in both groups were smaller than their MDCs and therefore failed to rule out the possibility of measurement variation due to error (Table 2).

**Table 3:** General Estimating Equations (GEE) results for outcome variables

Outcome Variables	B (95% CI)	*P*-value
CMMSE		
- Group	0.17 (− 0.47, 0.80)	0.607
- Time	0.10 (− 0.53, 0.74)	0.750
- Group* Time	1.33 (0.53, 2.13)	0.001*
IADL-CV		
- Group	0.51 (−0.20, 1.22)	0.160
- Time	−0.24 (− 0.85, 0.38)	0.445
- Group* Time	1.07 (0.34, 1.81)	0.004*

*p≤0.05

T0, baseline of the study; T1, 4 months’ post-intervention (T1 with the baseline [T0] as reference). Only the model estimates of regression coefficients (**B**) of the group (Group 0 = control [reference], I = intervention). Effect sizes: Chinese version of Mini-Mental State Examination (T1 vs T0), Cohen’s *d* = 0.50; Hong Kong Chinese version of Lawton Instrumental Activities of Daily Living scale (T1 vs T0), Cohen’s *d* = 0.48

*Abbreviations*: *CI* confidence interval, *CMMSE* Chinese version of Mini-Mental State Examination, *IADL-CV* Hong Kong Chinese version of Lawton Instrumental Activities of Daily Living scale

## Discussion

The key findings of current study suggested that elderly persons with MCI who practiced Tai Chi yielded significant improvement in general cognitive functions and IADL when compared to those participants with MCI without practicing Tai Chi, after adjusting the age difference, education level, marital status and living conditions for the two study groups, over the 16 weeks of the study period.

In the current study, CMMSE was used as baseline cognitive screening and subsequent cognitive assessment for all participants. There were several advantages for using CMMSE as a cognitive assessment tool. First, administration of the test took around 5 to 10 min for each participant, which was not overloaded for elderly participants. More importantly, MMSE has been used widely in clinical research to evaluate cognitive function and impairment. It facilitated comparisons and discussions of findings of the current study with previous cognitive research.

However, some researchers criticized that MMSE was not sensitive enough to distinguish different cognitive changes [54, 55], and even suggested that the Montreal Cognitive Assessment (MoCA) was a better choice for screening people with MCI [54]. The Hong Kong version of the MoCA was found to be reasonably good but not better than CMMSE in screening MCI [56]. Besides, using CMMSE as cognitive assessment would be easier for illiterate subjects since there was only one question related to verbal command. In addition, older illiterate respondents were only required to draw an overlapping pentagon in CMMSE, which was relatively easier than the clock drawing in the MoCA [56].

The results from this study confirmed the premise about the positive association between Tai Chi and global cognition in elderly persons with MCI. The findings supported previous studies of Tai Chi training in enhancing general cognitive functions of elderly persons with MCI [46, 57–60], as measured by MMSE. It was important to note that participants of the IG demonstrated significant improvement statistically after Tai Chi intervention on CMMSE and IADL-CV scores, when compared to the CG, but the scores changes did not exceed the limits of the respective MDCs in the current study. Such results indicated that the observed positive changes in CMMSE and IADL-CV scores were not large enough to rule out the possibility of measurement variation due to error. Larger scale studies are necessary to confirm the positive effects of Tai Chi on general cognition and IADL performance among elderly persons with MCI.

Tai Chi has been recognized as an alternate form of aerobic exercise, with a low-to-moderate intensity which is comparable to brisk walking [27]. In addition to aerobic efficacy, the meditation component of Tai Chi serves as a relaxation and stress reduction strategy, beneficent to preserve cognitive functions. As a cognition stimulating activity, practicing Tai Chi is found to improve attentional focus and executive function through learning series of movements in a continual sequence [30, 31]. Therefore, Tai Chi training can be expected to give rise to more significant improvements in cognitive functions, as compared to other types of physical exercise. An earlier randomized control trial by Lam et al. (2011, 2012) compared the effectiveness of Tai Chi versus stretching and toning exercise in the maintenance of cognitive abilities in older Chinese people with MCI. Significant improvements in CMMSE and other cognitive assessments on memory and executions were found in both group at the five-month follow-up, but not at the one-year follow-up. Compared to the stretching and toning exercise group, there were fewer subjects from the Tai Chi group who had progressed to clinical dementia at five-month and one-year follow-up. However, the protective effect of Tai Chi in delaying dementia could not be confirmed because the Tai Chi group was better educated and had better baseline cognitive functions than the stretching and toning exercise group [46, 58]. In another randomized control trial by Mortimer et al. (2012), 120 non-demented older community-dwelling subjects were randomized to one of four groups, walking exercise, Tai Chi, social interaction, and no intervention (control). After a 40-week intervention, the Tai Chi group showed significant improvement in several neuropsychological measures including the Mattis Dementia Rating Scale, representing general cognitive functioning. The social interaction group also showed enhancements in some, but fewer, neuropsychological measures. No difference was noted for the walking exercise and control groups on any neuropsychiatric measure across time [61]. This finding suggested that Tai Chi was the best strategies for preserving cognitive functions in older adults. However, the result needed to be interpreted with caution because the sample population was subdivided into around 30 subjects participating in each study group. As the sample size of this study was too small, the possibility of a Type II error could not be overlooked. Moreover, this study did not obtain sufficient statistical power to detect significant differences between study groups for neuropsychiatric variables.

Although the present study supported positive effects of Tai Chi on general cognition of older adults with MCI, there was no comparative exercise group, meaning that there was no provision to monitor the effects of Tai Chi relative to other types of physical exercise in enhancing general cognition of this population group.

There were vast differences in various cognitive studies in deciding the optimal dose and frequency of Tai Chi intervention. For the studies reported positive impacts on global cognitive functions, the durations of study interventions ranged from 14 [59] to 40 weeks [61] of regular instruction from a Tai Chi master. The frequencies of group sessions varied from once a week [46, 58] to three times a week [60, 61] and the session durations ranged from 40 [60] to 60 min [59]. In the present study, a 16-week short course of Tai Chi training was used, with participants meeting twice a week for 60 min per session, and this brought out positive changes in the global cognitive performances of this group of elderly persons with MCI. The implication of this clinical application can serve as a cost-effective cognitive health promotion program for the public.

Global cognitive status might be predictive of IADL performance in elderly persons [62, 63]. Functional evaluation of IADL performance has a central role in evaluating the progress of cognitive degeneration. However, there was limited empirical evidence to support the use of mind-body exercise interventions, to impact on IADL performance in people suffering from MCI. After 16 weeks of Tai Chi training, the IG demonstrated a statistically-significant improvement in IADL performance, measured by the IADL-CV, compared to the usual-care CG who had not been assigned any physical activity. This is the first study indicating that Tai Chi can be considered as an effective strategy in modifying IADL performance for elderly persons with MCI.

### Limitations

Although the results seemed promising, the study was limited by not having adopted a randomized control trial, by using convenience sampling, and by not making any attempt to blind the investigator and participants in the study, leading to the risk of assessor and respondent biases, and by not making follow-up arrangements. In the current study, the recruitment process started after center assignment. Participants knew exactly what their treatment would be before they joined the study. It could enhance their willingness to participate [64] while it might have increased the likelihood of self-selection bias. Next, it remains unclear whether Tai Chi provides equal or superior cognitive benefits compared to other moderate intensity aerobic exercises. Future large-scale, well-conducted randomized control trials with appropriate comparison groups and follow-up arrangements are required to evaluate the long-lasting effect of Tai Chi on cognitive health.

## Conclusion

Tai Chi is a mind-body therapy which comprises of both physical and intellectual components. It has been found to be a safe and culturally relevant exercise for elderly persons to practice. The results of this interventional study are promising, considering its short duration. The effects on general cognition and IADL performance in community-dwelling elderly persons with MCI are favorable.

## Additional file

Additional file 1: Table S1. The Yang-style simple form of Tai Chi (DOCX 14 kb)

## Abbreviations

BADL: Basic activities of daily living
CG: Control group
CI: Confidence interval
CMMSE: Chinese version of Mini-Mental State Examination
GEE: General Estimating Equations
IADL: Instrumental activities of daily living
IADL-CV: Hong Kong Chinese version of Lawton Instrumental Activities of Daily Living scale
IG: Intervention group
MCI: Mild cognitive impairment
