# Supplementary material for: Effects of tai chi on cognition and instrumental activities of daily living in community dwelling older people with mild cognitive impairment
Source: BMC Geriatr. 2018 Feb 2;18:37. doi: 10.1186/s12877-018-0720-8 (PMC5797349; doi:10.1186/s12877-018-0720-8)
Supplement: Additional file 1: Table S1. — The Yang-style simple form of Tai Chi (DOCX 14 kb) [file 12877_2018_720_MOESM1_ESM.docx]

|  | **Style Name** |
| --- | --- |
| 1. | Commencing Form起勢 |
| 2. | Parting Wild Horse’s Mane – Left, Right, Left左右野馬分鬃 |
| 3. | White Crane Flashing Wings白鶴亮翅 |
| 4. | Brush knee – Right, Left and Right左右摟膝拗步 |
| 5. | Playing the Lute手揮琵琶 |
| 6. | About Rewind Brachial (Repulse Monkey) 左右倒捲肱 |
| 7. | Stroking Bird’s Tail - Left左攬雀尾 |
| 8. | Stroking Bird’s Tail - Right右攬雀尾 |
| 9. | Single Whip單鞭 |
| 10. | Waving Hands like Clouds雲手 |
| 11. | Single Whip單鞭 |
| 12. | Pat on the High Horse高探馬 |
| 13. | Right Heel Kick右蹬腳 |
| 14. | Punching Ears with Both Fists雙峰貫耳 |
| 15. | Turn and Left Heel Kick轉身左蹬腳 |
| 16. | Lower Movement and Golden Cock Standing on One Leg-Left左下勢獨立 |
| 17. | Lower Movement and Golden Cock Standing on One Leg-Right右下勢獨立 |
| 18. | Fair Lady Works the Shuttles Left and Right左右穿梭 |
| 19. | Needle at Sea Bottom海底針 |
| 20. | Fan Back扇通臂 |
| 21. | Turn to Deflect Downward, Parry and Punch轉身搬攔捶 |
| 22. | Apparent Closing up如封似閉 |
| 23. | Cross Hands十字手 |
| 24. | Closing Form收式 |

**Style names are adapted from:** [**http://baike.baidu.com/view/390880.htm**](http://baike.baidu.com/view/390880.htm)
